# A prospective single‐center, single‐arm, open‐label, phase II study of sintilimab and anlotinib combined with chemotherapy in neoadjuvant treatment of resectable esophageal cancer

**DOI:** 10.1111/1759-7714.15312

**Published:** 2024-05-21

**Authors:** Hongtao Duan, Zhaoyang Wang, Lili Cao, Yifang Zhu, Liping Tong, Xiaolong Yan

**Affiliations:** ^1^ Department of Thoracic Surgery at Tangdu Hospital Air Force Medical University Xi'an China; ^2^ Department of Anesthesiology and Surgery at Tangdu Hospital Air Force Medical University Xi'an China

**Keywords:** antiangiogenic, chemotherapy, neoadjuvant treatment, resectable esophageal cancer, sintilimab

## Abstract

**Background:**

Antiangiogenic treatment and immunochemotherapy effectively treat patients with advanced esophageal cancer. However, there remains a dearth of studies concerning neoadjuvant therapy for resectable esophageal cancer.

**Methods:**

The study focused on patients with T2–4NxM0 resectable esophageal carcinoma. Neoadjuvant treatment involved administering anlotinib (10 mg orally, once a day, 2 weeks on and 1 week off) for antiangiogenesis and sintilimab (200 mg) and chemotherapy for three cycles. Surgical treatment was performed 4–6 weeks after the last chemotherapy cycle was completed. The primary endpoints assessed were pathological complete response (pCR) and safety.

**Results:**

Out of the 34 screened patients, 17 were successfully enrolled in the study, and 14 completed the entire treatment process. The pCR was 35.3% (6/17). However, two patients experienced mortality. The occurring rate of grade 3 or higher complications after the surgery was 78.6% (11/14) according to Clavien–Dindo classification. Specifically, anastomotic leakage was observed in 57.1% (8/14) of the patients.

**Conclusion:**

Compared to neoadjuvant chemotherapy, the current regimen demonstrated improved pCR. However, it did not show significant improvement compared to immunochemotherapy. It is essential to exercise caution when using this treatment approach in patients with esophageal cancer as it might increase postoperative complications, especially anastomotic leakage.

## Graphical abstract

This study focused on the neoadjuvant therapy of immune checkpoint (sintilimab) and receptor tyrosine kinase (anlotinib) inhibition combined with chemotherapy in resectable esophageal cancer. To the best of our knowledge, the first results of sintilimab and anlotinib combined with chemotherapy in neoadjuvant treatment of resectable esophageal cancer are reported. Out of the 34 screened patients, 17 were successfully enrolled in the study, and 14 completed the entire treatment process. The postoperative pCR rate was 35.3% (6/17 patients). However, two patients experienced death Clavien–Dindo classification identified the incidence of grade 3 or higher complications after the surgical treatment as 78.6% (11/14). Specifically, postoperative anastomotic leakage was observed in 57.1% (8/14) of patients. This finding suggests that compared to neoadjuvant chemotherapy, sintilimab and anlotinib combined with chemotherapy demonstrated improved postoperative pCR rates. However, it did not show significant improvement compared to immunochemotherapy. It is essential to exercise caution when using this treatment approach in patients with esophageal cancer as it might increase postoperative complications, especially anastomotic leakage.

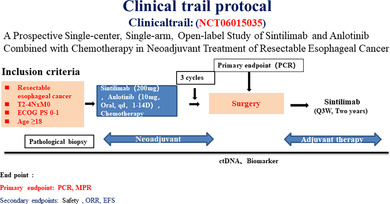

## INTRODUCTION

Esophageal cancer ranks sixth in incidence and eighth in mortality among malignant tumors.^1^ The National Comprehensive Cancer Network (NCCN) guidelines recommend neoadjuvant concurrent chemoradiotherapy combined with surgery as the optimal treatment modality for resectable esophageal cancer.^2^ Recently, there have been updates to the neoadjuvant combination therapy approach involving immune checkpoint inhibitors (ICIs), chemotherapy, and surgery, although it is considered a tertiary recommendation. Several studies have explored the efficacy of neoadjuvant ICIs combined with chemotherapy for resectable esophageal cancer, improving pathological complete response (pCR) in surgical patients.^3^, ^4^, ^5^, ^6^ However, the impact on overall survival (OS) remains unexplored, possibly due to time constraints. A recent study by Yang et al. retrospectively analyzed the results of neoadjuvant ICIs combined with chemotherapy for resectable esophageal cancer in China. This study included 370 patients.^7^ In the meta‐analysis by Ge et al., the pCR was 34.6%, inclusive of 25.8% ypT0N0 (no residual tumor and no affected lymph nodes) and 8.8% ypT0N+ (no residual tumor and no affected lymph nodes).^8^ This meta‐analysis encompassed 27 clinical studies, where the pCR was 31.4%. Subgroup analysis revealed a pCR of 32.4% for squamous cell carcinoma and 25.2% for adenocarcinoma. While neoadjuvant ICIs and chemotherapy has attained high pCR and has ensured safety, our treatment expectations remain unfulfilled. Hence, there is a need to explore and optimize better treatment options.

Antiangiogenic treatment works by normalizing the blood vessels within the tumor. Anlotinib, an antineoplastic agent, functions as a receptor tyrosine kinase (RTK) inhibitor with multiple targets, including VEGFR1, VEGFR2, VEGFR3, c‐KIT, PDGFRβ, and others, thereby displaying a potent antiangiogenic effect.^9^ A clinical study examining anlotinib in combination with toripalimab for second‐line treatment of esophageal cancer or borderline esophageal tumors showed that 29 patients achieved partial response (PR), with an overall response rate (ORR) of 32.3%. Furthermore, the use of next‐generation sequencing (NGS) in this study suggested that anlotinib induces changes in the tumor immune microenvironment, improving the treatment efficacy and implying that antiangiogenesis might enhance the effect of immunotherapy when used in combination by modulating the tumor immune microenvironment.^10^ Furthermore, in a study focusing on neoadjuvant treatment for resectable esophageal cancer, the addition of anlotinib and chemotherapy did not significantly differ in achieving pCR when compared to neoadjuvant concurrent chemoradiotherapy (10% vs. 7.7%).^11^ However, the incidence of adverse events (AEs) was lower in the anlotinib group than in the concurrent chemoradiotherapy group. Given the potential for combined antiangiogenic treatment regimens to revolutionize esophageal cancer treatment, a single‐center, open‐label, single‐arm study was conducted to investigate the efficacy of sintilimab and anlotinib combined with chemotherapy in the treatment of resectable esophageal cancer.

## METHODS

### Patient selection

The study was conducted from April 2021 to April 2022 and was approved by the Tangdu Hospital, Fourth Military Medical University (no. K202204‐02) and enrolled in clinicaltrials.gov (NCT06015035). Patient consent was acknowledged.

The inclusion criteria were as follows: patients aged 18–75 years with a histopathological diagnosis of esophageal cancer staged according to the AJCC eighth edition as T1–4N1–3M0. If patients were staged as T2N0M0, the esophageal lesions had to be ≥5 cm. Before enrollment, all patients underwent various examinations, including cardiac color ultrasound (left ventricular ejection fraction of at least 50%), pulmonary function (forced expiratory volume‐1 [FEV1] ≥1.5 L), enhanced chest computed tomography (CT), abdominal color ultrasound, cervical lymph node color ultrasound and other necessary laboratory tests (such as blood routine, liver and kidney function, electrolytes, and cortisol rhythm) to exclude treatment and surgical contraindications and ensure suitability for ICI treatment.

The exclusion criteria were as follows: Patients unable to tolerate surgery, those with refractory hypertension and proteinuria, those who had previously received other treatments, and those who were not suitable candidates for ICIs (due to conditions such as hepatitis B with viral quantification >2000 IU, systemic lupus erythematosus, and xerosis).

### Treatment regimen

Patients were treated with sintilimab (200 mg, day 1), anlotinib (QD, PO, Day 1‐14) combination with chemotherapy (albumin paclitaxel 130 mg/m^2^, day 1 and day 8 + nedaplatin 80 mg/m^2^, day 1) for three cycles of neoadjuvant therapy. Surgical intervention (Mckeown or Ivor Lewis approach) was performed 4–6 weeks after completing the last treatment. After the fourth week after surgery, patients received adjuvant therapy with sintilimab (200 mg, every 3 weeks) for 1 year. If any adverse reactions of grade 3 or higher occurred during the treatment, the dose of chemotherapeutic drugs was reduced by 25% until the patient's condition recovered to grade 1 or returned to normal, and the subsequent treatment cycle was continued. In cases of grade 3 or higher hypertension or proteinuria, the dose of anlotinib was reduced to 8 mg. It was discontinued if the condition could not recover to grade 1–2 after symptomatic treatment.

### Study endpoints

#### Primary endpoints

Pathological complete response was assessed by examining the postoperative pathological tissue for the absence of tumor cells in the primary tumor and lymph nodes.

Adverse reactions during neoadjuvant therapy were recorded following CTCAE version 5.0 guidelines. Perioperative complications were assessed using the Clavien–Dindo classification.

#### Secondary endpoints

(1) Major pathological response (MPR) refers to the proportion of residual tumor cells in the primary tumor and lymph nodes in the postoperative pathological tissue being <10%, or the primary tumor completely disappearing, and the number of positive lymph nodes being ≤1. (2) R0 resection rate: R0 resection was defined as achieving negative upper and lower resection margins. (3) Response evaluation criteria in solid tumors (RECIST) criteria assessment: Complete response (CR): complete response of target lesions; PR: >30% regression of target lesions; Non‐CR/non‐PD: target lesions did not completely disappear and did not increase by >20%, or other new lesions appeared in the body; Stable disease (SD): target lesions were reduced or increased by <20%; Progressive disease (PD): target lesions had increased by >20%.

### Statistical analysis

The sample size was determined using Simon's two‐stage design. With a minimum expected pCR of 20% and an expected pCR of 40%, a type I error (*α*) of 0.05, and a type II error of 80%, a sample size of 34 was calculated. In the first stage, 17 patients were enrolled. The study was carefully monitored to limit the number of pCR cases to three or below, and any increase in the risk of surgery and mortality due to the treatment regimen would have led to its discontinuation. All continuous variables are presented as frequencies. Statistical significance was set at *p* < 0.05.

## RESULTS

### Patient characteristics

**FIGURE 1:**
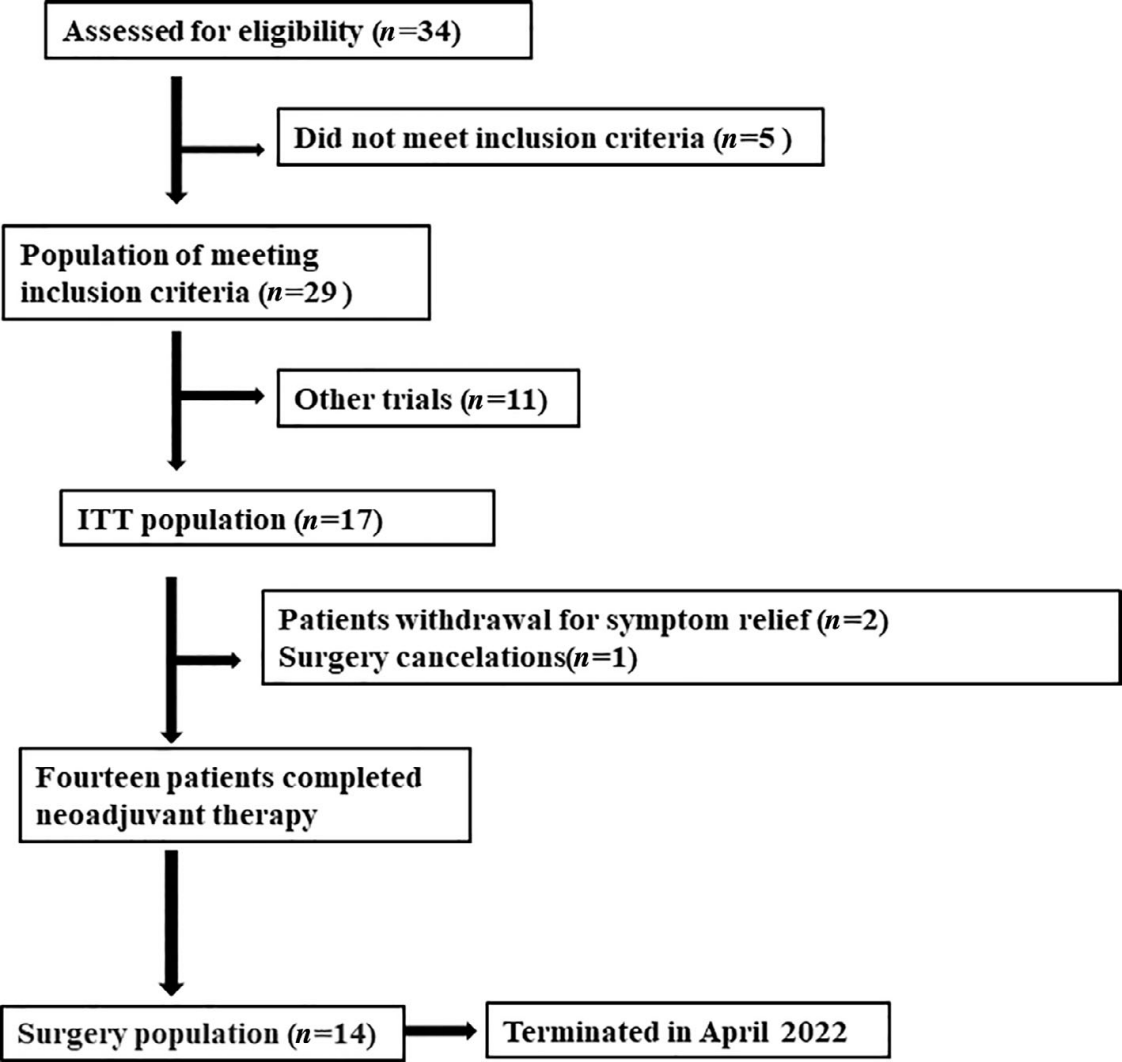
Patients enrolled in the study.

Between April 2021 and April 2022, 17 patients were enrolled in this study (Figure 1), with a mean age of 61.8 (44–74) years. Of the enrolled patients, 13 were men, and four were women. Five, nine, and three patients were diagnosed with clinical stages II, III, and IVA, respectively. Tumor location analysis showed one case in the upper segment, eight in the middle segment, and eight in the lower segment. Surgical treatment was performed in 14 patients, with four receiving it after completing two cycles of treatment, 10 of them after completing three cycles, and the remaining did not undergo surgical treatment (two patients experienced symptom relief, and one refused surgical treatment) (Table 1).

**TABLE 1:** Patient characteristics.

Age (years)	61.8 (44–74)
Sex	
Male	13
Female	4
BMI (kg/m^2^)	
≥24	7
>18 and <24	8
≤18	2
cT	
T2	2
T3	14
T4	1
cN	
N0	5
N1	7
N2	3
N3	2
Clinical stage	
II	5
III	9
IVA	3
Tumor site	
Upper	1
Middle	8
Distal	8

Abbreviation: BMI, body mass index.

### Efficacy

Following three treatment cycles and preoperative evaluation based on the RECIST criteria, six patients demonstrated CR, one showed PR, one had SD, and seven exhibited non‐CR/non‐PD, yielding an ORR of 41.2%. Among the 14 patients treated surgically, the postoperative pathological stages were distributed as follows: stage I (nine patients), stage II (two), stage IIIA (one), stage IIIB (one), and stage IVA (one). Pathological downstaging was observed in 10 patients (71.4%). pCR was achieved in six out of 17 patients (35.2%), and MPR was observed in eight (47.1%, 8/17) (Table 2).

**TABLE 2:** Efficacy.

RECIST	
CR	6
PR	1
SD	3
Non‐CR/Non‐PD	7
Pathological evaluation	
ypT	
T0	7
T1	2
T2	1
T3	3
T4	1
ypN	
N0	11
N1	1
N2	2
Yp stage	
I	9
II	2
IIIA	1
IIIB	1
IVA	1
Pathological downstaging	10
Endpoint	
pCR	6
MPR	8

Abbreviations: CR, complete response; MPR, major pathological response; pCR, pathological complete response; PD, progressive disease; PR, partial response; RECIST, response evaluation criteria in solid tumors; SD, stable disease.

**FIGURE 2:**
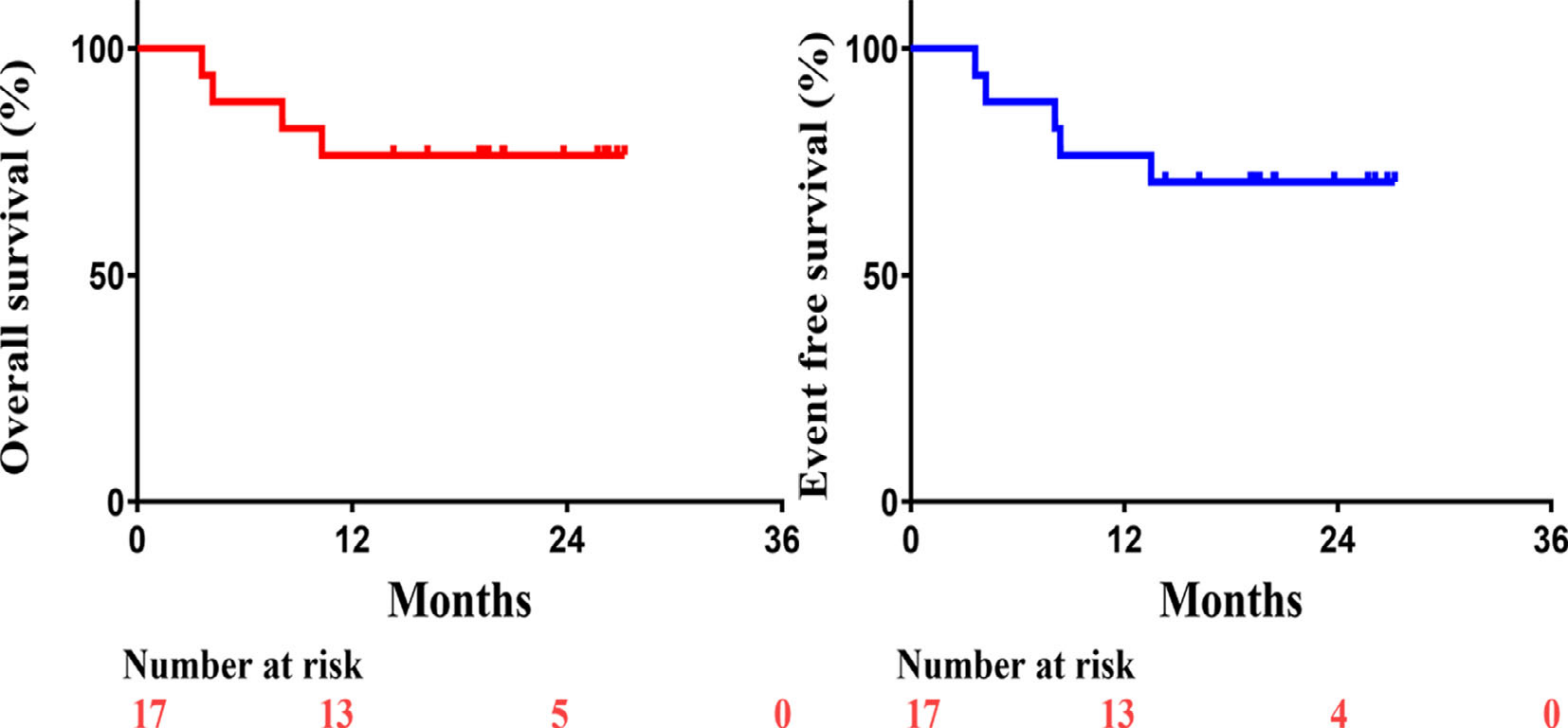
Kaplan–Meier curves of overall survival (left) and event‐free survival (right) in the modified intention‐to‐treat population (*n* = 17).

As of June 30, 2023, the median event‐free survival (EFS) and overall survival (OS) were not achieved. However, two patients died due to perioperative complications, one died 1 year after surgery, and another unoperated patient died from heart failure, resulting in a 1‐year overall survival of 76.5% (13/17 patients). Similarly, the 1‐year EFS was 76.5% (13/17) (Figure 2).

### Safety

Neoadjuvant AEs were recorded following CTCAE version 5.0. The incidence of grade 3 or higher was observed in eight patients, and one developed immune mucositis (stomatitis) due to sintilimab, which improved with hormone therapy, leading to the discontinuation of sintilimab. Other AE included leukopenia, neutropenia, nausea, and fatigue. Immune‐specific AE presented primarily as rashes in two patients (14.3%), classified as grade 1–2 (Table 3).

**TABLE 3:** Neoadjuvant adverse events.

	1–2 grade	≥3 grade
Febrile neutropenia	6	3
Anemia	7	1
Thrombocytopenic purpura	5	1
Nausea	6	2
Fatigue	4	1
Alopecia	3	1
Peripheral sensory neuropathy	4	1
Rash	2	0
Dry mouth	0	1

The incidence of postoperative complications was assessed using the Clavien–Dindo. According to the study design, two of 14 patients (14.3%) experienced grade five complications, while the other incidence of grade 3 or higher was observed in 11/14 patients (78.6%). Consequently, this clinical study was urgently terminated in April 2022. In particular, the incidence of anastomotic leakage was remarkably high at 57.1% (8/14 patients), with bronchopleural fistula occurring in two patients (14.3%), intrathoracic hemorrhage in two (14.3%) and acute respiratory distress syndrome (ARDS) in two (14.3%). Two patients died of intrathoracic hemorrhage on postoperative day 5 and ARDS 19 days after the surgical treatment, respectively (Table 4).

**TABLE 4:** Surgical complications.

	*N*	Clavien–Dindo (≥3)
Pneumonia	6	5
Acute respiratory distress syndrome	2	2
Hemorrhage	2	1
Anastomotic fistula	8	8
Bronchopleural fistula	2	2
Heart failure	6	0
Hypoalbuminemia	5	0
Hyponatremia	2	2
Anemia	3	0
Death	2	2
Adrenal insufficiency	2	0

## DISCUSSION

To the best of our knowledge, this is the first phase II clinical study to investigate the combination of neoadjuvant immuchemotherapy and anlotinib for resectable esophageal cancer. Although it was prematurely terminated because of the high incidence of grade 3 or higher postoperative complications and mortality, this study indicated potential limitations of this treatment regimen in esophageal cancer and was immediately decided to discontinue.

Although the pCR of this study (35.2%) was comparable to results from previous studies involving immunochemotherapy, the economic perspective raises concerns about using multiple antiangiogenic drugs in the regimen, especially when their efficacy is similar to immunochemotherapy.

Furthermore, the safety of the neoadjuvant regimens used in this study warrant more attention. The unexpectedly high incidence of anastomotic leakage (57.1%) in this study is a cause of concern. This percentage is significantly higher than the 0%–24% range reported in previous studies and even surpasses the incidence of postoperative anastomotic leakage currently observed in neoadjuvant immunochemotherapy.^12^ A comparative study between neoadjuvant chemotherapy combined with antiangiogenic therapy and exclusive neoadjuvant chemotherapy for resectable esophageal cancer revealed that the combined group exhibited delayed wound healing and an increased incidence of anastomotic stoma in patients. The incidence of anastomotic stoma was twice as high in the combination group compared to the control group (24% vs. 12%), suggesting that antiangiogenesis could contribute to the higher incidence of anastomotic leakage.^13^ However, in a phase II clinical study, there was only a slight decrease in postoperative complications after anlotinib combined with chemotherapy compared to concurrent chemoradiotherapy, with incidences of 7.5% and 12.8%, respectively.^11^ However, the incidence of anastomotic leakage was comparable between the two groups in this study. Therefore, no direct evidence indicates that this specific treatment directly increases the incidence of postoperative complications in patients.

The confounding factors that could influence the incidence of anastomotic leakage were reanalyzed. The parameters such as body mass index (BMI), drinking history, smoking history, and operation time of patients were comparable to those in a previous neoadjuvant therapy trial (although not included in this article). Hence, these factors were not considered. However, the timing of the last treatment and the operation might influence the occurrence of anastomotic leakage. A previous study has shown that antiangiogenic agents could have sustained effects on VEGF inhibition beyond 6 weeks after dosing.^14^ Only a few rectal cancer trials combining bevacizumab with neoadjuvant chemotherapy or chemoradiation have previously shown an increased incidence of postoperative complications.^15^ In this study, 14 patients underwent the operation within 6 weeks after the last oral administration of anlotinib, potentially impacting incision healing.^15^ Therefore, selecting a more appropriate operation time might help reduce the incidence of anastomotic leakage.

However, there were limitations to the present study. First, it was designed as a single‐arm, two‐phase study without randomized controlled trials, which might not truly reflect the effect of this treatment regimen. Second, the research was prematurely stopped due to the high incidence of grade 3 or higher postoperative complications and mortality, and further investigation is needed to evaluate the safety of this treatment approach.

In conclusion, to the best of our knowledge, the present study is the first to attempt neoadjuvant therapy for esophageal cancer using antiangiogenic therapy and immunochemotherapy. Although the pCR has shown improvement compared to chemotherapy alone, this enhancement is insignificant compared to immunochemotherapy. Moreover, there was a significant increase in postoperative complications. Therefore, caution should be exercised when using this regimen for esophageal cancer, and additional studies are required to verify its safety and efficacy.

## AUTHOR CONTRIBUTIONS

Xiaolong Yan, Hongtao Duan: Conception and design. Xiaolong Yan and Liping Tong, Administrative support. Hongtao Duan, Zhaoyang Wang, Lili Cao and Yifang Zhu: Provision of study materials or patients. Hongtao Duan, Zhaoyang Wang, Lili Cao, Yifang Zhu and Liping Tong: Collection and assembly of data. All authors were responsible for writing the manuscript and its final approval for publication.

## FUNDING INFORMATION

The present study was supported by grants from the National Natural Science Foundation of China (no. 82173252), and Miaozi Talent Fund of Tangdu Hospital of Air Force Military Medical University.

## CONFLICT OF INTEREST STATEMENT

The authors declare that they have no competing interests.

## ETHICS STATEMENT

The study was approved by the Ethics Committee of Tangdu Hospital, Fourth Military Medical University (no. K202204‐02) and enrolled in clinicaltrials.gov (NCT06015035).

## Data Availability

The datasets used and/or analyzed during the current study are available from the corresponding author on reasonable request.
